# Differential Cell Line Susceptibility to Crimean-Congo Hemorrhagic Fever Virus

**DOI:** 10.3389/fcimb.2021.648077

**Published:** 2021-03-24

**Authors:** Shiyu Dai, Qiaoli Wu, Xiaoli Wu, Cheng Peng, Jia Liu, Shuang Tang, Tao Zhang, Fei Deng, Shu Shen

**Affiliations:** ^1^ State Key Laboratory of Virology and National Virus Resource Center, Wuhan Institute of Virology, Chinese Academy of Sciences, Wuhan, China; ^2^ National Biosafety Laboratory, Wuhan Institute of Virology, Chinese Academy of Sciences, Wuhan, China

**Keywords:** CCHFV, cell line, susceptibility, permissive cell line, tissue origins

## Abstract

Crimean-Congo hemorrhagic fever (CCHF) is a severe tick-borne viral disease of global concerns due to the increasing incidence and lack of effective treatments. The causative agent, CCHF virus (CCHFV), has been characterized for years; however, its tropism in cell lines of different host and tissue origins remains unclear. This study characterized the susceptibility of 16 human and 6 animal cell lines to CCHFV. Increased viral load and viral nucleoprotein expression, and productive CCHFV replication were detected in human vascular (HUVEC), renal (SW-13 and HEK-293), hepatic (Huh7), and cerebral (U-87 MG) cell lines, which were considered CCHFV-highly permissive cell lines. Renal cell lines derived from monkey and dog could also support CCHFV replication. This study evaluated the susceptibility of different cell lines to CCHFV and identified CCHFV-permissive cell lines. Our findings raise concerns regarding the use of cell lines in *ex vivo* studies of CCHFV and may have important implications for further fundamental research, which would promote understanding of CCHFV pathogenesis and transmission, as well as benefit designing strategies for disease prevention and control.

## Introduction

Crimean-Congo hemorrhagic fever (CCHF) is a severe infectious disease across a vast geographic area in Asia, Africa, and Europe with fatality rates of 5–30% in humans (Spengler et al., 2018). The aetiological agent CCHF virus (CCHFV) is a highly pathogenic tick-borne virus belonging to the genus *Orthonairovirus* of family *Nairoviridae*. CCHF severity and CCHFV infection/exposure in humans have been described based on clinical findings and epidemiological studies (Bente et al., 2013; Ozsoy et al., 2015); however, the molecular mechanism underlying CCHF pathogenesis is not well described. CCHFV may be asymptomatic in animals (Spengler et al., 2016). To date, the disease development upon CCHFV infection has been demonstrated in only a subset of immunocompromised mice and a *cynomolgus macaque* model (Haddock et al., 2018; Mendoza et al., 2018). The limits of appropriate animal models, as well as the requirement for high biosafety level containment may slow down the progress in developing CCHFV anti-viral drugs and vaccines.

Suitable *in vitro* models for investigating CCHFV infection and replication may promote defining of the molecular mechanisms of pathogenesis and benefit the assessing of drug candidates for antiviral effects. Monocytes, endothelial cells, hepatocytes, and renal cells have been shown to be permissive to CCHFV (Connolly-Andersen et al., 2009; Bente et al., 2010; Peyrefitte et al., 2010; Connolly-Andersen et al., 2011; Rodrigues et al., 2012; Foldes et al., 2020). However, susceptibility differences to CCHFV in cell lines originating from these cell types, as well as from other human and/or animal tissues, have not been extensively described.

In the current study, we characterized the cell susceptibility of various cell lines to CCHFV, including 16 human cell lines derived from the brain, blood vessels, liver, kidney, uterus, immune system, lung, muscle, and skin and 6 animal cell lines derived from monkey, pig, dog, and hamster. Viral load in culture supernatants, viral antigen expression in cells, virus growth properties, and cell morphology changes were assessed following CCHFV infection.

## Materials and Methods

### Cells and Virus

Human rhabdomyosarcoma (RD) cells, human umbilical vein endothelial cells (HUVEC), human hepatocarcinoma (Huh7) cells, and epidermal carcinoma (A431) cells were grown in Dulbecco’s modified Eagle’s medium (DMEM) supplemented with 10% fetal bovine serum (FBS; Gibco, Grand Island, NY, USA). Baby hamster kidney (BHK-21) cells, human embryonic kidney (HEK-293) cells, human cervix adenocarcinoma (HeLa) cells, African green monkey kidney (Vero) cells, African green monkey kidney, clone E6 (Vero E6) cells, Madin-Darby canine kidney (MDCK) cells, U-87 MG, HMC3, HepG2, SH-SY5Y, MRC-5, PK-15, and DH82 cells were cultured in Eagle’s minimum essential medium (EMEM) supplemented with 10% FBS. Human lung carcinoma (A549) cells were cultured in F12 medium supplemented with 10% FBS. SW-13 cells were cultured in L-15 medium supplemented with 10% FBS. HULEC-5a cells were cultured in MCDB131 supplemented with 10% FBS. THP-1 and Raji cells were cultured in RPMI-1640 medium supplemented with 10% FBS. Huh7, HUVEC, A549, MRC-5, and PK-15 cell lines were obtained from the National Virus Resource Center (NVRC, Wuhan, China). RD, A431, BHK-21, HEK-293, HeLa, Vero, Vero E6, U-87 MG, HMC3, HepG2, SH-SY5Y, MDCK, DH82, SW-13, HULEC-5a, THP-1, and Raji cell lines were obtained from the American Type Culture Collection (ATCC; Manassas, VA, USA). All cell lines were tested negative for mycoplasma using EZ-PCR Mycoplasma test kit (Biological Industries, Kibbutz Beit Haemek, Israel). All cell line details are summarized in **Table 1**. All cell lines were grown at 37°C in a 5% CO2 incubator.

**Table 1:** Cell lines tested for CCHFV infection susceptibility.

Cell line	Source	Cell type	Tissue	The first round of infection			The second round of infection			Permissiveness to CCHFV^&^
				CCHFV NP expression	Viral loads (copies/mL)	Times of viral loads normalized to baseline	CCHFV NP expression	Viral loads (copies/mL)	Times of viral loads normalized to baseline	
***Human***										
SW-13	ATCC no. CCL-105	Epithelial	Kidney	+++	1.4 × 10^11^	4527	++	9.2 × 10^9^	289	Highly permissive
HEK-293	ATCC no. CRL-1573	Epithelial	Kidney	+++	2.0 × 10^10^	620	++	1.0 × 10^10^	331	Highly permissive
HeLa	ATCC no. CCL-2	Epithelial	Cervix	++	2.2 × 10^10^	688	++	3.4 × 10^9^	106	Permissive
Huh7	NVRC: IVCAS 9.005	Hepatoma	Liver	+++	1.0 × 10^11^	3169	+++	3.6 × 10^10^	1136	Highly permissive
HepG2	ATCC no. HB-8065	Epithelial	Liver	−	3.0 × 10^10^	943	+	3.4 × 10^10^	1065	Non-permissive
A549	NVRC: IVCAS 9.096	Epithelial	Lung	++	8.0 × 10^9^	252	+	1.1 × 10^9^	36	Permissive
MRC-5	NVRC: IVCAS 8.003	Fibroblast	Lung	++	1.9 × 10^10^	593	+	1.6 × 10^9^	49	Permissive
HULEC-5a	ATCC no. CRL-3244	Endothelial	Lung	+	2.2 × 10^10^	687	−	2.9 × 10^9^	93	Permissive
THP-1	ATCC no. TIB-202	Monocyte	Peripheral blood	−	1.6 × 10^10^	505	−	5.9 × 10^9^	186	Non-permissive
Raji	ATCC no. CCL-86	B lymphocyte	Lymphoblast	++	1.4 × 10^9^	440	+	2.7 × 10^9^	85	Permissive
U-87 MG	ATCC no. HTB-14	Epithelial	Brain	+++	4.1 × 10^10^	1297	++	1.6 × 10^10^	519	Highly permissive
HMC3	ATCC no. CRL-3304	Microglia	Brain	+	1.6 × 10^10^	322	+	4.2 × 10^9^	132	Permissive
SH-SY5Y	ATCC no. CRL-2266	Epithelial	Bone marrow	+	1.8 × 10^9^	57	−	1.4 × 10^9^	45	Permissive
RD	ATCC no. CCL-136	Rhabdomyosarcoma	Muscle	++	2.2 × 10^10^	682	++	1.6 × 10^10^	362	Permissive
HUVEC	NVRC: IVCAS 9.133	Endothelial	Umbilical	+++	7.1 × 10^9^	225	+++	2.7 × 10^9^	84	Highly permissive
A-431	ATCC no. CRL-1555	Epithelial	Skin/epidermis	−	1.7 × 10^10^	522	−	6.7 × 10^8^	21	Non-permissive
***Monkey***										
Vero	ATCC no. CCL-81	Epithelial	Kidney	+++	9.5 × 10^9^	298	+++	7.4 × 10^9^	232	Highly permissive
Vero E6	ATCC no. CRL-1586	Epithelial	Kidney	+++	1.9 × 10^10^	603	+++	3.4 × 10^10^	1078	Highly permissive
***Dog***										
DH82	ATCC no. CRL-10389	Macrophage	Macrophage	+++	1.7 × 10^11^	5215	++	6.5 × 10^10^	2043	Highly permissive
MDCK	ATCC no. CCL-34	Epithelial	Kidney	++	1.2 × 10^10^	372	++	3.5 × 10^9^	109	Permissive
***Pig***										
PK-15	NVRC: IVCAS 8.027	Epithelial	Kidney	++	1.5 × 10^10^	481	−	8.0 × 10^9^	253	Permissive
***Hamster***										
BHK-21	ATCC no. CCL-10	Fibroblast	Kidney	++	1.4 × 10^11^	431	−	6.8 × 10^9^	214	Permissive

CCHFV, Crimean-Congo hemorrhagic fever virus; ATCC, American Tissue Culture Collection; NVRC, National Virus Resource Center, Wuhan, China.

^&^Permissiveness to CCHFV was defined based on the viral loads of fold changes > 100 times in culture supernatants in addition to detectable NP expression.

−, the expression of CCHFV NP was undetectable; +, the expression of CCHFV NP was detected in a few of single cells or foci of less than three cells; ++, the expression of CCHFV NP was detected in confluent cells while NP-negative cells could also be observed in the dish; +++, the expression of CCHFV NP was detected in almost all cells and NP-negative cells could hardly be noted.

Crimean-Congo hemorrhagic fever virus (CCHFV) strain YL16070 (GenBank accession: KY354082) used in this study was originally isolated from *Hyalomma asiaticum* ticks by inoculating suckling mice with tick homogenates *via* both intracranial and intraperitoneal routes as previously described (Guo et al., 2017). A batch of CCHFV-positive brains from diseased mice was obtained from the National Virus Resource Center (IVCAS 6.6329) and homogenized in PBS using the Tissue Cell-Destroyer (NZK Biotech, Wuhan, China), followed by centrifugation at 5000 × g for 5 min to clarify cell debris. Then the clarified homogenates were filtered through a 0.22-μm filter, sub-packaged, and stored at -80°C until further experiment. All experiments using CCHFV were performed in a biosafety level 3 laboratory at Wuhan Institute of Virology, Chinese Academy of Sciences.

### Virus Infection Assay

Viral titer of CCHFV in filtered supernatant from mouse brain homogenates was determined using end-point dilution assay as described previously (Zhang et al., 2018). Briefly, Vero E6 cells were cultured in 96-well plates to 50% confluence and infected with ten-fold serial dilutions of the supernatants. At 5 days post-infection (d p.i.), cells were fixed and evaluated by immunofluorescence assay (IFA) to determine the viral titers (TCID_50_/mL). Viral titer endpoints were calculated using the Reed–Muench method.

Cells (5 × 10^5^ cells per well) were seeded in 6-well plates one night before the infection assay. Briefly, the growth medium was discarded and the cells infected with 10 μL cleared supernatant from mouse brain homogenates (diluted to 500 μL/well with appropriate medium containing 2% FBS) at a multiplicity of infection (MOI) of 0.01. After 1 h of viral adsorption at 37°C, the cells were supplemented with 1500 μL/well of appropriate medium containing 2% FBS and then incubated at 37°C in a 5% CO_2_ atmosphere. This infection was designated as the first round of infection. Supernatants of the first round of infection were collected at 4 d p.i. based on the growth property of CCHFV on Vero cells in the previous study (Zhang et al., 2018), and were clarified by centrifuge (5000 × g for 5 min). A 1 mL aliquot of clarified supernatant of the first round of infection from each cell line was used to inoculate fresh cells (37°C for 1 h) followed by supplemented to 2 mL with fresh growth medium, which was designated as the second round of infection. Supernatants at 4 d p.i. were also collected and clarified as mentioned above. All supernatants were frozen at −80°C until Real-time reverse transcription polymerase chain reaction (qRT-PCR) analysis. The infected cells from the first and second rounds of infection were fixed and evaluated by IFA to analyze CCHFV nucleoprotein (NP) expression in the cells.

### Detection of CCHFV Infection

Real-time reverse transcription polymerase chain reaction (qRT-PCR) analysis was performed to determine viral loads. Total RNA was extracted from culture supernatants of all 22 cell lines infected by CCHFV at 4 d p.i. using TRIzol reagent (Takara, Kusatsu, Japan) according to the manufacturer’s instructions. One-step qRT-PCR was used to determine the viral loads using a One Step PrimeScript™RT-PCR Kit (Takara) and a StepOnePlus Real-Time PCR System (Applied Biosystems, Foster City, CA, USA). The primers, probes, and RNA standards were prepared as previously described (Zhang et al., 2018). The viral RNA copies in the diluted supernatant that was prepared from mouse brain homogenates and inoculated with cells was defined as the viral load baseline. To evaluate the efficiency of virus proliferation from each cell line, viral loads in supernatants from infected cells were normalized to the baseline as fold changes.

The expression of CCHFV NP in cells at 4 d p.i. was detected by IFA as previously described (Guo et al., 2017). Based on the expression of NP, the cell lines were classified into 3 levels (−, the expression of CCHFV NP was undetectable; +, the expression of CCHFV NP was detected in a few of single cells or foci of less than three cells; ++, the expression of CCHFV NP was detected in confluent cells while NP-negative cells could also be observed in the dish; +++, the expression of CCHFV NP was detected in almost all cells and NP-negative cells could hardly be noted).

For some cells, the expression of NP was further quantified by flow cytometry using a BD LSRFortessa flow cytometer (BD Biosciences). Briefly, infected or mock-infected cells were detached with trypsin at 2 d p.i., and fixed with 4% paraformaldehyde in phosphate-buffered saline (PBS). The fixed cells were incubated in 0.2% Triton X-100-PBS for permeabilization and then blocked with 5% bovine serum albumin (BSA). The cells were then stained with fluorescein isothiocyanate (FITC)-conjugated CCHFV NP monoclonal antibody (NZK Biotech, Wuhan, China) at room temperature for 1 h. The cells were washed three times with PBS containing 0.5% BSA prior to analysis.

### Growth Curve Analyses

Viral growth properties of CCHFV in different cell lines were characterized by growth curve analysis. Briefly, cells (5 × 10^5^) were seeded in the 6-well plates overnight and were then incubated with the filtered supernatant from mouse brain homogenates at an MOI of 0.01. After 1 h of adsorption at 37°C, the cells were washed three times with PBS and the appropriate medium was added. Cells were then incubated for the indicated time at 37°C. Supernatants (50 μL) were harvested from each sample at the indicated time points and used to determine the viral titers by end-point dilution assays as described above.

### Electron Microscopy

Vero E6 cells were infected with CCHFV at an MOI of 0.01. The culture supernatants (20 mL) were collected at 4 d p.i., inactivated with β-propiolactone (1:2000 v/v), cleared of cell debris and concentrated to 1 mL using Amicon Ultra concentrators (Millipore, Billerica, MA, USA). The clarified supernatants were then centrifuged at 13000 × g for 30 min at 4°C. The pellets were resuspended in 10 μL PBS. Virus suspension were adsorbed onto formvar-coated copper grids for 5 min, negatively stained using 2% phosphotungstic acid (PTA) for 1 min and then examined with a transmission electron microscope (H-7000 FA; Hitachi, Japan).

## Results

**Figure 1:**
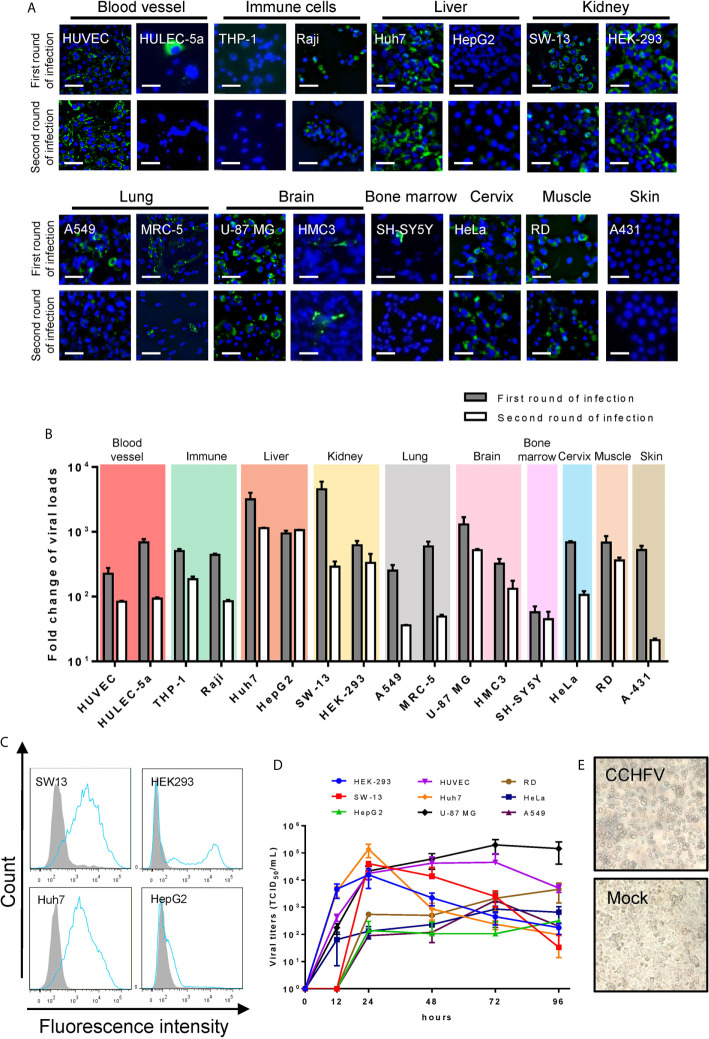
Human cell line susceptibility to CCHFV. **(A)** Human cell line susceptibility to CCHFV as determined by immunofluorescence assay (IFA). Cells were infected with CCHFV at an MOI of 0.01 and CCHFV nucleoprotein (NP) expression was detected at 4 days post-infection (d p.i.) using IFA. Infected cells exhibited green fluorescence (NP). Bars, 30 μm. **(B)** Human cell line susceptibility to CCHFV as defined by fold change of viral loads. Various cell lines were infected with CCHFV at an MOI of 0.01. Supernatants were harvested at 4 d p.i. and used for qRT-PCR. All experiments were performed in duplicate. Fold-change of viral loads were normalized to baseline viral load. **(C)** Flow cytometric analysis of CCHFV-infected cells. Different cell lines were infected with CCHFV at an MOI of 0.01. Cells were harvested at 2 d p.i. and expression of CCHFV NP was detected using fluorescein isothiocyanate (FITC)-conjugated CCHFV NP monoclonal antibody and analyzed using flow cytometry. The shaded area represents the control. The mean florescence intensity (MFI) of mock-infected/CCHFV-infected were 837/10200, 44/6491, 179/5528 and 375/839 for SW13, HEK293, Huh7 and HepG2 cells respectively. At least 10000 cells were used for analysis. **(D)** Viral characteristics of CCHFV in 6 human cell lines. The different cell lines were infected with CCHFV at an MOI of 0.01. Supernatants were harvested at the indicated time points post infection. Viral titers in the supernatants were determined using end-point dilution assays. Means ± SD of three represented independent experiments are shown. **(E)** SW-13 cells were infected with CCHFV at an MOI of 0.01. Cytopathic effects of cell rounding and detachment were evaluated at 4 d p.i. using an inverted microscopy. Mock, uninfected SW-13 control.

CCHFV infection in each cell line was characterized by IFA. During the first round of infection, 14 of the 16 human cell lines were infected with CCHFV at different efficiencies, including HUVEC, HULEC-5a, Raji, Huh7, HepG2, SW-13, HEK-293, A549, MRC-5, U-87 MG, HMC3, SH-SY5Y, HeLa and RD (**Figure 1A** and **Table 1**). To reflect the infectivity of progeny virus generated in the supernatants from the first round of infection, 1 mL of cell debris-clarified supernatant was used to inoculate with fresh cells for the second round of infection. During the second round of infection, the expression of NP was also detected in 12 cell lines, including HUVEC, Raji, Huh7, HepG2, SW-13, HEK-293, A549, MRC-5, U-87 MG, HMC3, HeLa, and RD (**Figure 1A**).

Viral loads varying from 1.8 × 10^9^ to 1.4 × 10^11^ copies/mL were detected in culture supernatants of all the 16 human cell lines in the first round of infection. These levels were 50–4,000 times higher than the baseline viral load of 3.2 × 10^7^ copies/mL (**Figure 1B** and **Table 1**), suggesting virus replication in these cell lines. High viral loads exceeding 1,000-fold increases above baseline were detected in supernatants of Huh7, SW-13, and U-87 MG cells, while viral loads less than 100 times that of baseline were observed in SH-SY5Y cells. Viral titers in supernatants from HUVEC, HeLa, and HepG2 were determined at 96 hours post infection (h p.i.) which showed that higher viral titers were generated from HUVEC cells (3.2 × 10^4^ TCID_50_/mL) than those from HeLa (3.2 × 10^3^ TCID_50_/mL) and HepG2 (4 × 10^2^ TCID_50_/mL). When the supernatant was used to inoculate fresh cells for the second round of infection, CCHFV RNA was detected in supernatants from all cell lines, but the levels were significantly reduced except for HepG2 cells having comparable virus loads between the first and second rounds (**Figure 1B**).

Based on the viral loads in supernatants and viral NP expression in cells during the first round of infection, we defined CCHFV-permissive cell lines as having viral loads of fold changes > 100 times in culture supernatants in addition to detectable NP expression (**Table 1**). Of the 16 tested human cell lines, HUVEC, Huh7, SW-13, HEK-293, and U-87 MG were CCHFV-highly permissive cell lines; HULEC-5a, Raji, A549, MRC-5, HMC3, SH-SY5Y, HeLa, and RD were CCHFV-permissive cell lines; THP-1, HepG2, and A431 were CCHFV-non permissive cell lines (**Table 1**). HepG2 and HEK-293 cells, which are derived from the same tissues as Huh7 and SW-13 cells, respectively, showed different susceptibility to CCHFV infection. Especially for HepG2 cell, the infection efficiency of CCHFV was lower than that in Huh7 cell. This was further supported by quantitative analyses using flow cytometry (**Figure 1C**). CCHFV growth properties were subsequently characterized by determining infectious progeny virus yields in supernatants of CCHFV-highly permissive cell lines (HUVEC, Huh7, SW-13, U-87 MG, and HEK-293), CCHFV-permissive cell lines (A549, HeLa, and RD), and CCHFV-non permissive cell line (HepG2) to better illustrate CCHFV permissiveness to different cell lines. Virus titers from each cell line increased rapidly during the initial 24 h p.i. with the highest titer (1.4 × 10^5^ TCID_50_/mL) generated in Huh7 cells (**Figure 1D**). After 24 hours of infection, virus yields were sustained in supernatants of HUVEC, U-87 MG, A549, HeLa, RD and HepG2 cells, but decreased in supernatants of SW-13, HEK-293, and Huh7 cells. At 72 h p.i., virus titers in supernatants from HUVEC, U-87 MG, A549, and HeLa cells arrived peak with the highest titer (2.0 × 10^5^ TCID_50_/mL) generated in U-87 MG cells. However, viral titers in supernatants from RD and HepG2 cells still increased slightly from 72 h p.i. to 96 h p.i. Virus titers in supernatants from CCHFV-non permissive cell line HepG2 was only around 3.2 × 10^2^ TCID_50_/mL, but that from CCHFV-highly permissive cell lines (HUVEC, Huh7, SW-13, HEK-293, and U-87 MG) could reach at least 10^4^ TCID_50_/mL and from CCHFV-permissive cell lines (A549, HeLa, and RD) could reach at least 10^3^ TCID_50_/mL (**Figure 1D**). In addition, SW13 cells showed the most prominent cytopathic effects (CPE) including cell rounding and detachment (**Figure 1E**).

**Figure 2:**
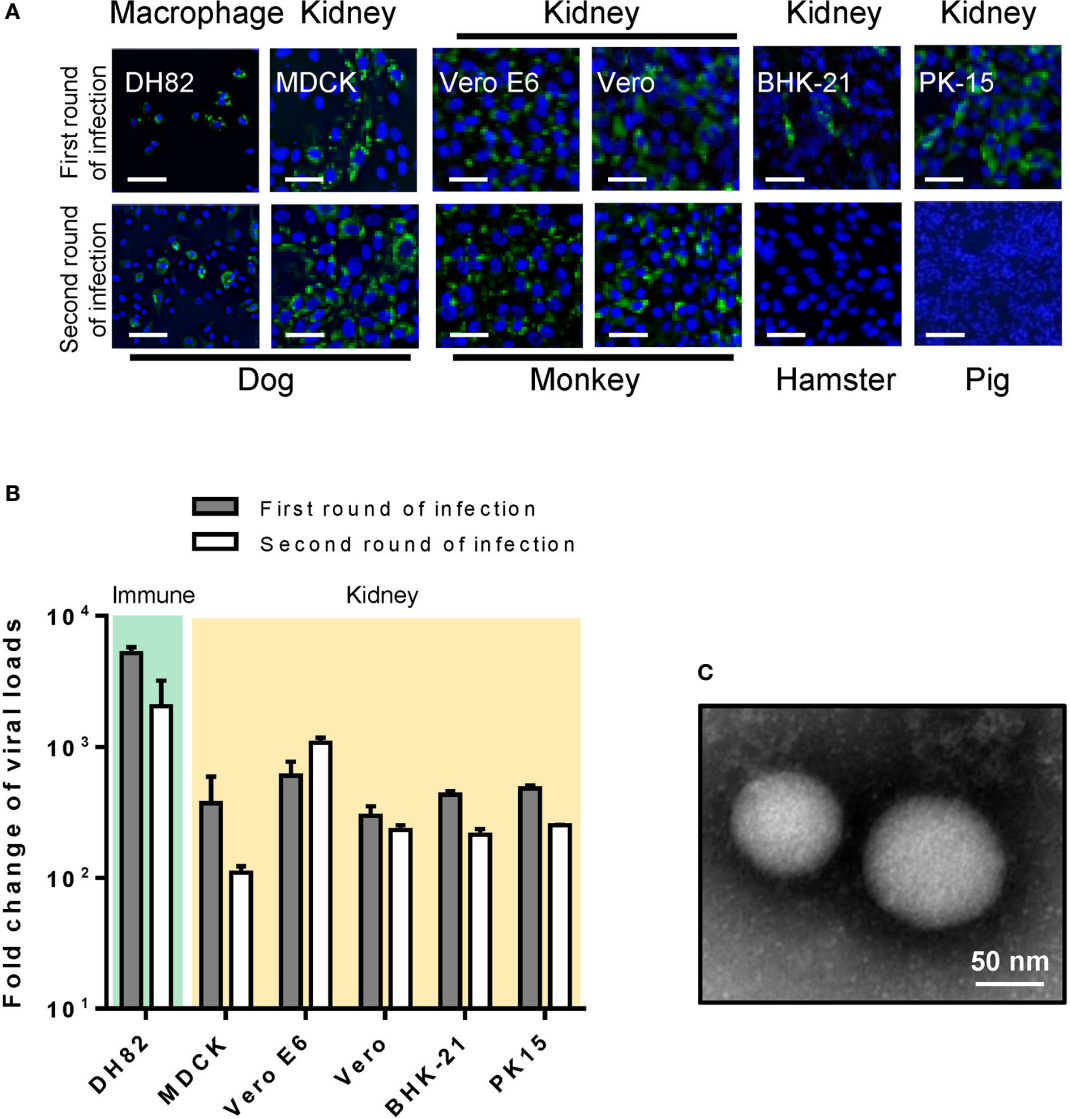
Animal cell line susceptibility to CCHFV. **(A)** Animal cell line susceptibility to CCHFV was determined by IFA. Cells were infected with CCHFV at an MOI of 0.01 and CCHFV NP expression was detected at 4 d p.i. using IFA. Infected cells exhibited green fluorescence (NP). Bars, 30 μm. **(B)** Animal cell line susceptibility to CCHFV as defined by fold-change of viral loads. The different cell lines were infected with CCHFV at an MOI of 0.01. Supernatants were harvested at 4 d p.i. and evaluated using qRT-PCR. All experiments were performed in duplicate. Fold-change of viral loads were normalized to baseline viral load. **(C)** Electron micrographs of negative staining of CCHFV particles in cell culture supernatants from Vero E6. Bar, 50 nm.

CCHFV susceptibility was characterized for six animal cell lines. During the first round of infection, CCHFV infection was detected in all animal cell lines using IFA (**Figure 2A**) and viral loads in supernatants from different cell lines were 200–5,000 times greater than baseline (**Figure 2B** and **Table 1**). Similar to that in human cell lines, when the supernatant was used to inoculate fresh cells for the second round of infection, CCHFV infection could be detected in all the 6 cell lines with decreased infection efficiency except for Vero E6 cell (**Figure 2B**). CCHFV virions having typical bunyavirus morphology of spherical and enveloped particles with a diameter of 80-100 nm were observed from culture supernatants of Vero E6 (**Figure 2C**), further suggesting sustainable CCHFV proliferation in Vero E6 cell line. Of the 6 tested animal cell lines, DH82, Vero E6 and Vero were CCHFV-highly permissive; MDCK, BHK-21 and PK-15 were CCHFV-permissive cell lines (**Table 1**).

## Discussion

CCHFV is one of the prioritized pathogens requiring urgent research of the World Health Organization due to its risk to public health and national security (Mehand et al., 2018); however, knowledge about its pathogenesis are very limited. Studies of cell susceptibility to CCHFV is limited although a wide range of cells originating from human and animals has so far been demonstrated to be susceptible to CCHFV (Connolly-Andersen et al., 2009; Connolly-Andersen et al., 2011; Rodrigues et al., 2012; Spengler et al., 2017; Foldes et al., 2020). In this study, we characterized viral load, viral antigen expression, virus growth properties, and CPE in 22 cell lines derived from different tissues during CCHFV infection, for a comparative assessment of the cell susceptibility to CCHFV.

The cell line susceptibilities to CCHFV were summarized in **Table 1** based on viral loads in culture supernatants and NP expression in cells during the first round of infection. Due to the limited residual brain homogenate in culture supernatants during the first round of infection, in addition to newly amplified virus, the supernatants also contained remaining progenitors. To compare the efficiency of virus proliferation between different cell lines, we defined the initial viral RNA copies in mouse brain homogenates used to inoculate with cells as viral load baseline and viral RNA copies in cell lines were normalization against it. Among these cell lines, HUVEC, Huh7, SW-13, HEK-293, U-87 MG, DH82, Vero E6 and Vero were CCHFV-highly permissive; HULEC-5a, Raji, A549, MRC-5, HMC3, SH-SY5Y, HeLa, RD, MDCK, BHK-21 and PK-15 were CCHFV-permissive cell lines; THP-1, HepG2, and A431 were CCHFV-non permissive cell lines. To reflect the infectivity of progeny virus generated in the supernatants from each cell line, 1 mL of the collected culture supernatants were also used to infect new cells for the second round of infection. However, viral loads and the expression of NP decreased in most cell lines (**Figures 1** and **2**). This could be correlated to the viral titers generated from the first round of infection resulting in comparatively low MOIs used for second round of infection (MOIs for HUVEC: 6.4 × 10^-2^, HeLa: 6.4 × 10^-3^, and HepG2: 8 × 10^-4^). In addition, the different IFN-competence of each cell line may also be involved. Previous studies have shown that CCHFV is a potent inducer of the interferon response and upregulate the interferon signaling pathway in A549, Huh7, HepG2, Vero cells and primary macrophages (Andersson et al., 2008; Peyrefitte et al., 2010; Papa et al., 2015; Kozak et al., 2020). The RNA-seq data of HUVEC and SW-13 cells, which are highly permissive to CCHFV, showed CCHFV infection induced robust IFN response (unpublished data). Since the old medium transferred from the first round of infection were not removed from the second round of infection, a number of factors, such as interferon and cell degradations, may be present in supernatants of second infection, which may affect the infection efficiencies.

**Figure 3:**
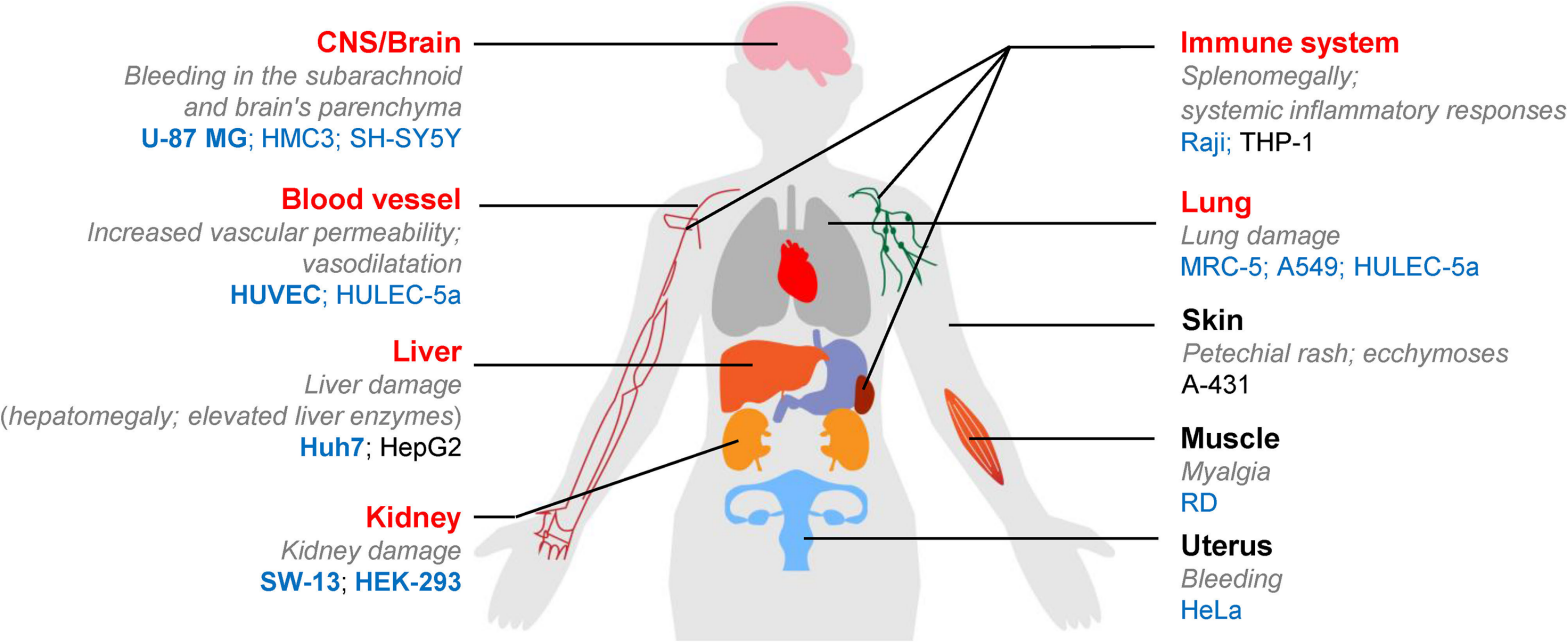
Graphic representation summarizing the correlation between CCHFV susceptibility of human cell lines tested in the current study and tissue origins as well as respective clinical manifestations in human body. The main targeting tissues of CCHFV are indicated in red font as previously reported (Akinci et al., 2013; Ozsoy et al., 2015). The main clinical manifestations in the organs or systems are presented in grey italics. CCHFV-permissive human cell lines were indicated by blue font as defined in our current study. The five cell lines (U-87 MG, HUVEC, Huh7, HEK293 and SW-13) of high susceptibility to CCHFV as suggested for the use of *ex vivo* fundamental studies are highlighted in bold. Non-permissive cell lines are shown in black.

CCHFV causes severe peripheral circulation viraemia, hemorrhage, and tissue lesions, ultimately resulting in multi-organ failure. It initially replicates in blood, liver, and spleen and then systemically spreads to kidney, brain, and lung (Bente et al., 2010; Akinci et al., 2013; Ozsoy et al., 2015; Haddock et al., 2018). To better understand the differential susceptibilities of cell lines to CCHFV, we summarized tissue origins of the human cell lines used in this study in correlation with the tissue targets for CCHFV infection and respective clinical manifestations. As shown in **Figure 3**, the tissue tropisms of CCHFV were consistent with the origins of most permissive cell lines. Vascular endothelial cells (HUVEC) are susceptible to CCHFV, which could be activated to increase vascular permeability and initiates inflammatory responses (Connolly-Andersen et al., 2011). The monocyte cell lines THP-1 could not support CCHFV replication well, probably because virus replication in monocytes is controlled by an efficient interferon-induced response (Connolly-Andersen et al., 2009; Peyrefitte et al., 2010). CCHFV infects hepatocyte cells, which induces ER-stress and triggers apoptosis (Rodrigues et al., 2012). The high susceptibility of the human hepatocyte cell line Huh7 to CCHFV could correlate with the salient clinical features of hepatic injury (Ergonul et al., 2006; Bente et al., 2013). In contrast, the hepatoma cell line HepG2 was unable to sustain efficient CCHFV proliferation. We speculate that HepG2 cells provided CCHFV RNA transcription and replication as high viral loads were detected in supernatants, but produced defective virus particles as indicated by very few infected cells and reduced yields of infectious viruses. This was also suggested by calculating viral RNA copies over TCID_50_, which showed that HepG2 cells generated virus of 7.5× 10^7^ copies to induce infection of 1 TCID_50_, while these for HUVEC and HeLa were 2.28 × 10^5^ copies/TCID_50_ and 7.05× 10^6^ copies/TCID_50_, respectively. Huh7 and HepG2 cells respond differentially to CCHFV, which was supported by the different gene expression in HepG2 and Huh7 cells infected with CCHFV according to RNA-Seq (Kozak et al., 2020). The IFN status of cell lines may correlate with the permissiveness of CCHFV. Huh7 cells in lack of IFN-β expression (Li et al., 2005) could better support CCHFV replication than the IFN-β competent HepG2 cells. Previous studies demonstrated that viral replication occurs in adrenal gland and kidney (Bente et al., 2010; Haddock et al., 2018), which was consistent with our findings of CCHFV tropism in renal cells (SW-13 and HEK-293). CCHFV infection inducing CPE in SW-13 cells may trigger a host-encoded necrotic program as anti-CCHFV response, as viral CPE may be largely attributed to host defenses and viral anti-defenses but not directly coupled to viral reproduction (Agol, 2012). Central nervous system (CNS) infection and neurological involvement have been reported in CCHF (Spengler et al., 2017). In our study, the neuroblastoma cell line SH-SY5Y was non-permissive to CCHFV infection, while the glial cell lines U-87 MG and HMC3 responsible for immune surveillance in brain were susceptible to CCHFV infection. Cerebral hemorrhage observed in clinical cases (Ozsoy et al., 2015) may be not attributed to CCHFV invasion of neurocytes but increased vascular permeability. CCHFV is unable to spread *via* the respiratory tract. The lung-derived epithelial cell line A549, and fibroblast deriving MRC-5 cells were permissive to CCHFV, but infection was limited.

CCHFV growth kinetics were characterized and are distinguishable between the CCHFV-highly permissive cell lines (HUVEC, Huh7, SW-13, HEK-293, and U-87 MG) and CCHFV-permissive cell lines (A549, HeLa, and RD) as well as CCHFV-non permissive cell line (HepG2). Virus titers from all tested cell lines increased rapidly at the initial 24 hours of infection. The highly-permissive cell lines had comparatively titers of 1.8 × 10^4^ to 1.4 × 10^5^ TCID_50_/mL significantly higher than those of the non-permissive and permissive cell line (1.4 × 10^2^ to 5.4 × 10^2^ TCID_50_/mL). Viral titers generated from highly-permissive cell lines decreased significantly after 24 h, which may be related to IFN level and virus degradation over time. Decrease of viral titers was also observed in the permissive cell lines after 72 h, but was not significant probably due to the low yields of infectious viruses. Therefore, the IFN production and competence of these cell lines in response to CCHFV infection need to be further evaluated systematically so as to clarify the effect on the amplification of CCHFV. Meanwhile, the decrease of virus titers from the cell lines after 72 h would also explain the reduced infection as evidenced by IFA assays in the second round of infection. Therefore, we recommend harvesting virus culture supernatants at 24 or 48 h post infection for better use of the viruses in further *in vitro* assay with the highly-permissive cell lines like SW13, HEK293, and Huh7.

CCHFV infects various domestic livestock and wild animals. Most remain asymptomatic, even though they may develop severe viraemia (Spengler et al., 2016). CCHFV infection in renal cell lines derived from dogs, monkeys, hamsters, and pigs, although with varied susceptibilities, suggest these animals may serve as reservoirs and play a role in CCHFV dissemination. Tick cell lines and animal cell lines derived from major hosts of *Hyalomma* ticks, including cattle, sheep, and camels were not investigated. Further studies on cell lines derived from these and other animals may shed light on whether these animals serve as potential reservoirs, and provide an *ex vivo* approach for investigating the molecular mechanisms of infection in animal hosts.

There are limitations in this study and factors that may affect the results. First, the CCHFV strain YL16070 used in our study was isolated from *Hyalomma asiaticum* ticks in China. It is phylogenetically divergent from the prototype strain IbAr10200 which was isolated from *Hyalomma excavatum* ticks in Nigeria (Sanchez et al., 2002; Guo et al., 2017). Based on geographic distributions, YL16070 strain belongs to Asia groups and IbAr10200 strain belongs to Africa group (Guo et al., 2017). The evolutionary differentiation of these two strains may suggest amino acid variations of viral proteins and different infectivity. Besides, in the virus life cycle, the use of different principal vector tick hosts and virus-amplifying vertebrate species in the different geographic regions may reflect difference in cell line susceptibility (Sanchez et al., 2002). However, we failed to compare susceptibility of these two strains due to the lack of strain IbAr10200. Second, the growth kinetics could only present CCHFV growth during the conventional amplification process. Because the growth kinetics was determined using a low amount of viruses like 0.01 MOI in this study, which is commonly used for virus amplification, whereas of the high amount of viruses like 5 MOI was commonly used for one-step growth curve analysis. This was because the substantial titer of CCHFV (5 × 10^5^ TCID_50_/mL) in the mouse brain homogenate was insufficient to support the infection assays with high dose of viruses. In the growth curves, CCHFV titer was measured with TCID_50_, but not PFU, which may also cause different understanding of infectivity. Third, despite the cell lines were obtained from ATCC, the different batches of the cell lines in individual laboratories may also affect the efficiency of detection.

This study compared the susceptibility to CCHFV in human and animal cell lines derived from different tissues. The findings provide an important reference for the use of cell lines in *ex vivo* studies and highlight the need for careful consideration based on their host and tissue origin, necessity of CPE observation, and other characteristics that may be involved. Taken together, cell lines of vascular, hepatic, renal, and cerebral origins, which represent the targeting tissues of CCHFV invasion *in vivo*, exhibited high viral load in supernatants. Based on our findings, we suggest that U-87 MG, SW-13, HEK293, Huh7, and HUVEC are useful for investigating CCHFV pathogenesis and evaluating the effects of antiviral drugs specific to respective tissues, and Vero E6 is applicable for virus isolation and culture as sustainability of CCHFV proliferation. In summary, our findings provide information to be considered for optimizing *ex vivo* experiments for fundamental studies on CCHFV and developing CCHF disease control and prevention strategies.

## Data Availability Statement

The raw data supporting the conclusions of this article will be made available by the authors, without undue reservation.

## Author Contributions

SS and FD conceived and designed the study. SD, QW, XW, and CP performed the experiments. SD, QW, and SS analyzed the data. SD drafted the manuscript. SS, FD, JL, ST, and TZ reviewed and edited the manuscript. SS, FD, and ST acquired the funding. All authors contributed to the article and approved the submitted version.

## Funding

This work was supported by the National Program on Key Research Project of China (2018YFE0200402, 2019YFC1200700), the National Science and Technology Major Project on Important Infectious Diseases Prevention and Control (2018ZX10734-404), and the Strategic Biological Resources Capacity Building Project of Chinese Academy of Sciences (KFJ-BRP-017-06).

## Conflict of Interest

The authors declare that the research was conducted in the absence of any commercial or financial relationships that could be construed as a potential conflict of interest.

## References

[B1] Agol V. I. (2012). Cytopathic effects: virus-modulated manifestations of innate immunity? Trends Microbiol. 20, 570–576. 10.1016/j.tim.2012.09.003 23072900 PMC7126625

[B2] Akinci E. Bodur H. Leblebicioglu H. (2013). Pathogenesis of Crimean-Congo hemorrhagic fever. Vector Borne Zoonotic Dis. 13, 429–437. 10.1089/vbz.2012.1061 23663164

[B3] Andersson I. Karlberg H. Mousavi-Jazi M. Martinez-Sobrido L. Weber F. Mirazimi A. (2008). Crimean-Congo hemorrhagic fever virus delays activation of the innate immune response. J. Med. Virol. 80, 1397–1404. 10.1002/jmv.21222 18551619

[B4] Bente D. A. Alimonti J. B. Shieh W. J. Camus G. Stroher U. Zaki S.. (2010). Pathogenesis and immune response of Crimean-Congo hemorrhagic fever virus in a STAT-1 knockout mouse model. J. Virol. 84, 11089–11100. 10.1128/JVI.01383-10 20739514 PMC2953203

[B5] Bente D. A. Forrester N. L. Watts D. M. Mcauley A. J. Whitehouse C. A. Bray M. (2013). Crimean-Congo hemorrhagic fever: History, epidemiology, pathogenesis, clinical syndrome and genetic diversity. Antiviral Res. 100, 159–189. 10.1016/j.antiviral.2013.07.006 23906741

[B6] Connolly-Andersen A. M. Douagi I. Kraus A. A. Mirazimi A. (2009). Crimean Congo hemorrhagic fever virus infects human monocyte-derived dendritic cells. Virology 390, 157–162. 10.1016/j.virol.2009.06.010 19570561

[B7] Connolly-Andersen A. M. Moll G. Andersson C. Akerstrom S. Karlberg H. Douagi I.. (2011). Crimean-Congo hemorrhagic fever virus activates endothelial cells. J. Virol. 85, 7766–7774. 10.1128/JVI.02469-10 21632768 PMC3147921

[B8] Ergonul O. Celikbas A. Baykam N. Eren S. Dokuzoguz B. (2006). Analysis of risk-factors among patients with Crimean-Congo haemorrhagic fever virus infection: severity criteria revisited. Clin. Microbiol. Infect. Off. Publ. Eur. Soc. Clin. Microbiol. Infect. Dis. 12, 551–554. 10.1111/j.1469-0691.2006.01445.x 16700704

[B9] Foldes K. Aligholipour Farzani T. Ergunay K. Ozkul A. (2020). Differential Growth Characteristics of Crimean-Congo Hemorrhagic Fever Virus in Kidney Cells of Human and Bovine Origin. Viruses 12, 685. 10.3390/v12060685 PMC7354505 32630501

[B10] Guo R. Shen S. Zhang Y. Shi J. Su Z. Liu D.. (2017). A new strain of Crimean-Congo hemorrhagic fever virus isolated from Xinjiang, China. Virol. Sin. 32, 80–88. 10.1007/s12250-016-3936-9 28251517 PMC6598972

[B11] Haddock E. Feldmann F. Hawman D. W. Zivcec M. Hanley P. W. Saturday G.. (2018). A cynomolgus macaque model for Crimean-Congo haemorrhagic fever. Nat. Microbiol. 3, 556–562. 10.1038/s41564-018-0141-7 29632370 PMC6717652

[B12] Kozak R. A. Fraser R. S. Biondi M. J. Majer A. Medina S. J. Griffin B. D.. (2020). Dual RNA-Seq characterization of host and pathogen gene expression in liver cells infected with Crimean-Congo Hemorrhagic Fever Virus. PloS Neglected Trop. Dis. 14, e0008105. 10.1371/journal.pntd.0008105 PMC7162549 32251473

[B13] Li K. Chen Z. Kato N. Gale M. Lemon S. M. (2005). Distinct Poly(I-C) and Virus-activated Signaling Pathways Leading to Interferon-β Production in Hepatocytes. J. Biol. Chem. 280, 16739–16747. 10.1074/jbc.M414139200 15737993

[B14] Mehand M. S. Al-Shorbaji F. Millett P. Murgue B. (2018). The WHO R&D Blueprint: 2018 review of emerging infectious diseases requiring urgent research and development efforts. Antiviral Res. 159, 63–67. 10.1016/j.antiviral.2018.09.009 30261226 PMC7113760

[B15] Mendoza E. J. Warner B. Safronetz D. Ranadheera C. (2018). Crimean-Congo haemorrhagic fever virus: Past, present and future insights for animal modelling and medical countermeasures. Zoonoses Public Health 65, 465–480. 10.1111/zph.12469 29676526 PMC7165601

[B16] Ozsoy S. Gokmen A. Ozdemir M. Akduman B. Korkusuz I. Javan G. T. (2015). Medical examiners and Crimean-Congo hemorrhagic fever contamination risk. J. Forensic Leg Med. 36, 32–36. 10.1016/j.jflm.2015.08.010 26367781

[B17] Papa A. Mirazimi A. Koksal I. Estrada-Pena A. Feldmann H. (2015). Recent advances in research on Crimean-Congo hemorrhagic fever. J. Clin. Virol. 64, 137–143. 10.1016/j.jcv.2014.08.029 25453328 PMC4346445

[B18] Peyrefitte C. N. Perret M. Garcia S. Rodrigues R. Bagnaud A. Lacote S.. (2010). Differential activation profiles of Crimean-Congo hemorrhagic fever virus- and Dugbe virus-infected antigen-presenting cells. J. Gen. Virol. 91, 189–198. 10.1099/vir.0.015701-0 19812268

[B19] Rodrigues R. Paranhos-Baccala G. Vernet G. Peyrefitte C. N. (2012). Crimean-Congo hemorrhagic fever virus-infected hepatocytes induce ER-stress and apoptosis crosstalk. PloS One 7, e29712. 10.1371/journal.pone.0029712 22238639 PMC3253088

[B20] Sanchez A. J. Vincent M. J. Nichol S. T. (2002). Characterization of the glycoproteins of Crimean-Congo hemorrhagic fever virus. J. Virol. 76, 7263–7275. 10.1128/JVI.76.14.7263-7275.2002 12072526 PMC136317

[B22] Spengler J. R. Estrada-Pena A. Garrison A. R. Schmaljohn C. Spiropoulou C. F. Bergeron E.. (2016). A chronological review of experimental infection studies of the role of wild animals and livestock in the maintenance and transmission of Crimean-Congo hemorrhagic fever virus. Antiviral Res. 135, 31–47. 10.1016/j.antiviral.2016.09.013 27713073 PMC5102700

[B23] Spengler J. R. Kelly Keating M. Mcelroy A. K. Zivcec M. Coleman-Mccray J. D. Harmon J. R.. (2017). Crimean-Congo Hemorrhagic Fever in Humanized Mice Reveals Glial Cells as Primary Targets of Neurological Infection. J. Infect. Dis. 216, 1386–1397. 10.1093/infdis/jix215 28482001 PMC5853341

[B21] Spengler J. R. Bente D. A. Bray M. Burt F. Hewson R. Korukluoglu G.. (2018). Second International Conference on Crimean-Congo Hemorrhagic Fever. Antiviral Res. 150, 137–147. 10.1016/j.antiviral.2017.11.019 29199036 PMC6497152

[B24] Zhang Y. Shen S. Fang Y. Liu J. Su Z. Liang J.. (2018). Isolation, Characterization, and Phylogenetic Analysis of Two New Crimean-Congo Hemorrhagic Fever Virus Strains from the Northern Region of Xinjiang Province, China. Virol. Sin. 33, 74–86. 10.1007/s12250-018-0020-7 29520745 PMC6178084

